# How Does Commute Time Affect Labor Supply in Urban China? Implications for Active Commuting

**DOI:** 10.3390/ijerph17134631

**Published:** 2020-06-27

**Authors:** Xiaoyu Wang, Jinquan Gong, Chunan Wang

**Affiliations:** 1 National School of Development, Peking University, Beijing 100871, China; xiaoyuwangecon@pku.edu.cn (X.W.); jinquang@pku.edu.cn (J.G.); 2 School of Economics and Management, Beihang University, Beijing 100191, China

**Keywords:** commute time, labor supply, endogeneity, active commuting, China

## Abstract

This paper identifies the causal effect of commute time on labor supply in urban China and provides implications for the development of active commuting. Labor supply is measured by daily workhours, workdays per week and weekly workhours, and city average commute time is adopted as an instrumental variable to correct the endogenous problem of individual commute time. We find that in urban China, commute time does not have effect on daily labor supply but has negative effects on workdays per week and weekly labor supply. These results are different from those found in Germany and Spain, and are potentially related to the intense competition among workers in the labor market of China. Moreover, the effect of commute time on workdays per week is stronger for job changed workers. In addition, the effects of commute time on labor supply are not different between males and females. Finally, policy implications for active commuting are discussed.

## 1. Introduction

Active transportation and commuting are efficient ways to improve individuals’ physical activities, ameliorate individuals’ health status, and reduce air pollution. In recent years, many big cities in China, such as Beijing, have built a large number of exclusive bike lanes and encourage people to go to work using this healthy and green method. In fact, active commuting does not merely relate to health and the environment, but might also have a close relationship with economic activities. Compared with traditional urban transportation modes, such as the subway, buses, and cars, active commuting will inevitably increase individuals’ commute time. It has been shown that commute time might significantly affect labor supply [1,2,3]. Accordingly, if commute time is proved to have negative effects on labor supply, a potential conflict between active commuting and economic development may arise, which might threaten the development of active commuting to some extent. In this case, in order to better benefit from the functions of active commuting and transportation, remedies should be considered to mitigate the negative effects.

In this paper, we aim to identify the causal effect of commute time on labor supply in urban China, and provide policy implications for the promotions of active commuting.

There are divergent theoretical views on how to model the relationship between commuting and labor supply in the literature. Some studies assume that the number of workdays is fixed and that the number of workhours per day can be chosen [4], while other studies make the opposite assumption [5]. Therefore, valid empirical evidence of the causal effect of commuting on labor supply will shed some lights on this theoretical debate. Previous research has found that, in Germany, commute distance has a positive effect on daily and weekly labor supply but no effect on the number of workdays [1]. It has also been found that commute distance has a negative effect on productivity measured by worker’s absenteeism in Germany [2]. In Spain, it was found that longer commute time results in workers providing more daily labor supply [3]. It has been shown that when workers are free to make decisions on their daily labor supply but subject to daily exogenous variation in commute time, commute time has a negative effect on job-offer acceptance decisions of substitute teachers in Michigan [6].

In our study, labor supply is measured by daily workhours, workdays per week, and weekly workhours. These three measurements of labor supply are necessary since they capture different relationships between commute time and labor supply. Specifically, for a large proportion of workers, commute time is the fixed cost for daily workhours, while it is the variable cost for weekly workhours because workers can choose the number of workdays [4,5,7]. Using the China Health and Nutrition Survey (CHNS), and combining the fixed effect model and instrumental variable method, we find that commute time does not have a significant effect on daily workhours in China, which is in contrast with the findings in Germany and Spain [1,3]. On the other hand, commute time significantly decreases the number of workdays per week and weekly workhours. In addition, heterogeneous effects of commute time are found between job changed and job unchanged workers. Finally, policy implications for the development of active commuting are discussed.

This paper contributes to the literature by comparing the heterogeneous impacts of commute time on labor supply in China and other countries (i.e., Germany and Spain). The patterns observed in urban China, namely, that longer commute time does not significantly affect daily workhours but significantly decreases the number of workdays per week and weekly workhours, might closely relate to the intense competition among workers in the labor market of China. Such a particularity of China’s labor market implies that in order to better promote active commuting, the Chinese government should pay more attention to the amelioration of transportation infrastructure.

The remainder of this paper is organized as follows. The next section discusses the theoretical model established in this study, provides detailed information on the data employed, and introduces the identification strategy and the specification of the model. Section 3 presents empirical results and additional results. Section 4 discusses the reasons for different results across countries and policy implications for the development of active commuting. The final section concludes.

## 2. Methods

### 2.1. The Theoretical Model

Previous theoretical research presents a model of monocentric cities with decentralized employment [8]. This model proposes if the work location is assumed to be fixed, longer commuting will be compensated by lower house prices, or by higher wages when residential location is assumed to be fixed. Although the effect of commuting on labor supply can be analyzed based on White’s model, a large amount of the literature casts doubt on the ability of the monocentric model to explain actual commuting behavior in the US [9,10,11,12].

The model we set up in this paper does not assume that the city is monocentric and that work places are located in the center of city. On the contrary, work locations can be altered if workers change their jobs. Since the data we use is longitudinal, in which people surveyed stay in the same residential location, we assume that people do not change their residential locations. Hourly wage is a function of distance from residential location to work location, that is, w(d), in which w is the hourly wage and d is the round-trip commute distance.

Assume that the individual utility function is given by U(x,l), and that a worker wants to maximize utility subjected to their budget constraint:

(1) $$\begin{array}{c} \underset{x,l}{\max}U\left(x,l\right)\\ s.t.x+w\left(d\right)l=I_{0}+w\left(d\right)-\left(tw\left(d\right)+m\right)d, \end{array}$$

in which x is consumption; l is leisure, $I_{0}$ is non-labor income; t is the commute time for each unit of commute distance; td is the round trip commute time; and m the is the monetary cost of each unit of commute distance. The budget constraint can be rewritten as:

(2) $$x=I_{0}+w\left(d\right)-\left(tw\left(d\right)+m\right)d-w\left(d\right)l.$$

Putting the above specification into the utility function and maximizing the utility function:

(3) $$U_{l}-U_{x}w=0.$$

The optimal values of x and l are then as follows:

(4) $$x^{*}=x\left(w\left(d\right),I_{0}+w\left(d\right)-\left(tw\left(d\right)+m\right)d\right),$$

(5) $$l^{*}=l\left(w\left(d\right),I_{0}+w\left(d\right)-\left(tw\left(d\right)+m\right)d\right).$$

We assume that all workers have the same utility level; differentiate with respect to distance (d) and apply the envelope theorem:

(6) $$w_{d}=\frac{tw+m}{1-td-l}=\frac{tw+m}{n},$$

in which n=1−td−l is the labor supply. $w_{d}>0$ implies that higher wage compensates longer commute distance or commute time.

First, the marginal effect of commute distance on labor supply is:

(7) $$\frac{\partial n}{\partial d}=-t-\frac{\partial l}{\partial d},$$

in which:

(8) $$\begin{array}{l} \;\frac{\partial l}{\partial d}=\left(\frac{\partial l^{h}}{\partial w}-\frac{\partial l}{\partial m}l\right)w_{d}+\frac{\partial l}{\partial m}\left(w_{d}-\left(tw+m\right)-tdw_{d}\right)\\ =\frac{\partial l^{h}}{\partial w}w_{d}+\left(1-td-l\right)\frac{\partial l}{\partial m}w_{d}-\frac{\partial l}{\partial m}\left(tw+m\right)\\ =\frac{\partial l^{h}}{\partial w}w_{d}+\frac{\partial l}{\partial m}\left(nw_{d}-\left(tw+m\right)\right)\\ =\frac{\partial l^{h}}{\partial w}w_{d}. \end{array}$$

Note that the superscript “h” denotes Hicksian. Combining the two equations above, the marginal effect of commute distance on labor supply will be:

(9) $$\frac{\partial n}{\partial d}=-t-\frac{\partial l^{h}}{\partial w}w_{d}.$$

According to economic theory, given the individual’s utility is unchanged, the increase of hourly wage will substitute leisure time, that is, $\frac{\partial l^{h}}{\partial w}<0$. Considering also $w_{d}>0$, if the absolute value of $\frac{\partial l^{h}}{\partial w}$ or $w_{d}$ is large enough, the commute distance will have a positive effect on labor supply, that is, $\frac{\partial n}{\partial d}>0$. Furthermore, given constant commute time per unit of commute distance, commute time increases labor supply. However, if the absolute value of the product is not large enough, the commute distance will decrease labor supply, that is, $\frac{\partial n}{\partial d}<0$.

In addition, the change of commute time per unit of commute distance t can be understood as the variation of transport condition in an urban area. Then, the marginal effect of transportation condition on labor supply is given by:

(10) $$\frac{\partial n}{\partial t}=-d-\frac{\partial l}{\partial t}=-d+\frac{\partial l}{\partial I}dw=d\left(-1+\frac{\partial l}{\partial I}w\right),$$

in which I is used to denote the total income of a worker, that is, $I_{0}+w\left(d\right)-\left(tw\left(d\right)+m\right)d\equiv I$. Due to the positive income effect, the increase of total income will increase the leisure time, that is, $\frac{\partial l}{\partial I}>0$. Under this specification, when $\frac{\partial l}{\partial I}$ or w is large enough, the commute time per unit of commute distance or the deterioration of transportation condition will have a positive effect on labor supply, that is, $\frac{\partial n}{\partial t}>0$. Therefore, commute time will have a positive effect on working hours given that commute distance is held constant. Nevertheless, when the product of $\frac{\partial l}{\partial I}$ and w is small, the deterioration of transportation condition will then decrease labor supply.

To summarize, the derivation of theoretical model leads to the following propositions.

**Proposition** **1.** *The change of commute distance, such as induced by job change, has a positive effect on the wage, but its effect on labor supply is uncertain.*

**Proposition** **2.** *Better transportation conditions do not affect the wage, and its effect on labor supply is uncertain.*

### 2.2. Data and Descriptive Statistics

The dataset adopted in this study was drawn from the China Health and Nutrition Survey (CHNS) provided by the Chinese Center for Disease Control and Prevention and the Population Research Center of the University of North Carolina in the USA. The first round was in 1989. The next eight waves followed in 1991, 1993, 1997, 2000, 2004, 2006, 2009, and 2011. The study population is composed of respondents from the provinces of Guangxi, Guizhou, Heilongjiang, Henan, Hubei, Hunan, Jiangsu, Liaoning, and Shandong in China. This sample is diverse, with variation found in a wide-ranging set of socioeconomic factors, which include income, employment, education, and modernization and other related health, nutritional and demographic measures. The detailed information on commute time and other data at both individual and household levels make it ideal for examining the effect of commute time on labor supply.

To take advantage of controlling individual-specific, time-invariant effects, attention was restricted to a longitudinal subsample in the dataset. We further limited the sample to workers aged between 16 and 60 years who were surveyed in the urban areas. Note that urban areas in this paper include all urban, suburban, and town communities in the CHNS. For the purpose of this study, we also limited the sample to surveys after 2004 since CHNS only included questions on commute time for workers from 2004. We defined three variables relating to labor supply in this study, that is, workhours per day, workdays per week, and weekly workhours.

In line with the previous studies on labor supply, some variables reflecting socioeconomic and demographic characteristics of the respondents were specified. As illustrated in numerous economics studies, an individual’s status relating to smoking and chronic conditions is a signal of health and affects that individual’s labor supply decision, and was thus included in the labor supply function. In addition to health status, other demographic characteristics involved in the empirical analysis included age, the number of children less than or equal to 6 years old, and the number of family members in the household. In order to capture the wealth effect on labor supply, we also controlled for family wealth in the regression. Family wealth included house value and values of appliances, vehicles, machines, and equipment owned. Family income affects individual labor supply and thus was also controlled in our analysis. Family income, in this study, was defined as household non-labor income, other family members’ labor income, and housing subsidy, in which housing subsidy was calculated by subtracting the annual rent the family pays from the annual fair market rent.

To correct the endogenous problem of individual commute time, we used the average commute time in the city, which was constructed as the average of individual workers’ commute times in the city, as the instrumental variable for individual commute time. To avoid the problem that city characteristics may be potentially correlated with city average commute time and individual labor supply, we controlled for city-level human capital, measured by city average education level, and the ratio of employment from the non-public sector, as a variable from the labor demand side. We further controlled the fraction of workers changing job in the city to reflect the city labor market condition.

Finally, after excluding those observations with relevant missing information, a total sample of 1077 individuals and 2727 observations was retained. The descriptive statistics of commute time, labor supply variables, and other important variables in 2004, 2006, 2009 and 2011 are presented in Table 1.

On average, round trip commute time increased from 29.17 min in 2004 to 36.09 min in 2009 and then decreased to 34.68 min in 2011. All three labor supply variables decreased in 2006 first and then increased. For example, typical workers worked 8.05 h per day, 5.51 days per week, and 44.56 h per week in 2004. In 2006, daily working hours decreased to 7.93, working days decreased to 5.43, and weekly working hours also decreased. However, from 2009 to 2011, people worked longer per day, more days per week, and also had higher weekly working hours.

Family wealth and family income had a similar increasing trend. The proportion of smoking people was consistently around 33% across different years. The number of children aged less than 6 years in the household was around 0.14–0.17 and the number of family members in the household was around 3.2. The percentage of job changing workers ranged from 11% to 16%. Average years of education in the city was around 11 years. The ratio of non-public employment increased from 29% in 2004 to 44% in 2011.

### 2.3. Empirical Methodology

In this paper, we aim to investigate the causal effect of commute time on labor supply, measured by working hours per day, number of workdays per week, and weekly workhours. However, following the standard labor supply literature, controlling commute time, hourly wage, and other variables in regressions cannot provide a consistent estimator for such a causal effect, due to the following two reasons.

First, commute time is endogenous, especially when residential location and work location can be chosen by individual workers and such a decision is made based on the working hours. For example, long workhours may cause workers to move to places closer to the work places. In this research, worker’s residential location is assumed to be fixed in the longitudinal data. However, there are still two factors accounting for the change of commute time, that is, the changes of work location and transportation conditions in the city. As a result, the reverse causal effect of labor supply on work location decision cannot be ruled out, resulting in the endogeneity problem. Nevertheless, since city transportation conditions provide a good instrumental variable for commute time, we adopted the average commute time in the city to measure the city transportation conditions. Note that as the city average commute time is an aggregate variable, it can at least partially correct the endogeneity of individual commute time. From this perspective, although the city average commute time is not flawless, it might work as a valid instrumental variable to some extent.

Second, wages may also be endogenous. Given the residence location, workers can find jobs based on job characteristics, such as daily workhours, number of workdays per week, total weekly workhours, hourly wage, and commute time. Labor supply, wage, and commute time are simultaneously chosen. In this research, as hourly wage was not the variable of interest, in order to avoid the endogeneity problems, a totally reduced form was adopted first, and hourly wage was not included in the regression. Hourly wage was included in a subsequent regression to perform a robustness check.

The basic regression form is:

(11) $$\log\left(Y_{it}\right)=\beta_{0}+\beta_{1}\log\left(T_{it}\right)+\beta_{2}X_{it}+v_{i}+\mu_{t}+u_{it},$$

in which $Y_{it}$ measures labor supply variables, that is, daily workhours, working days per week, and weekly workhours; $T_{it}$ is the round trip commute time; $X_{it}$ are other time-varying independent variables, controlling for individual, household, demographic, and city characteristics; $v_{i}$ is the individual time-invariant unobserved heterogeneity; $\mu_{t}$ captures the year fixed effect; and $u_{it}$ is the error term. $\beta_{1}$ is the elasticity of labor supply with respect to commute time. The fixed effect is the model that fit using the above equation.

The first-stage regression for commute time is:

(12) $$\log\left(T_{it}\right)=\alpha_{0}+\alpha_{1}\log({\bar{T}}_{j})+\alpha_{2}X_{it}+v_{i}+\mu_{t}+\varepsilon_{it},$$

in which ${\bar{T}}_{j}$ is the average commute time in city *j* where individual *i* lives, and $\varepsilon_{it}$ is the error term. We also applied a fixed effect model to the above equation.

Note that by using the modified Wald test and the Cumby–Huizinga test, we found that the problems of heteroscedasticity and autocorrelation in the error terms exist. Therefore, in all regressions, we adjusted standard errors by clustering around individuals.

## 3. Results

### 3.1. First-Stage Results for Individual Commute Time

To validate the use of city average commute time as a good instrumental variable for individual commute time, two conditions need to be checked. First, the city average commute time should have a strong correlation with individual commute time. Second, the city average commute time should not correlate with the error term in Equation (11).

Table 2 presents regression results when the dependent variable is individual commute time, and independent variables are city average commute time and those which we controlled in the labor supply regression. Column (1) of Table 2 shows that city average commute time, that is, city transportation conditions, has a strong relationship with individual commute time. The elasticity is 0.875, which means that a 1% increase in city average commute time will enhance individual commute time by 0.875%. Controlling city level characteristics additionally, the correlation is still significant at the 1% level, which is shown in column (2) of Table 2. In labor supply regressions in the second stage, hourly wage was controlled. Thus, in order to show that city average commute time is still a good instrumental variable, we controlled the hourly wage in the first stage and present results in column (3) of Table 2. We find that there still exists a strong correlation between individual commute time and city average commute time, given other independent variables and hourly wage.

Results in Table 2 support that there is a significantly positive correlation between city average commute time and individual commute time. City average commute time reflects the transportation conditions in the city. Individual commute time is the time workers spend between residential location and work location, which should be affected by the city transportation conditions. A city with high average commute time has poor transportation conditions, which increase the commute time for commuters.

In addition, city average commute time is a macro condition in the city. Thus, it does not correlate with the factors that affect an individual’s labor supply and appears in the error term in Equation (11). 

Finally, in the last three rows of Table 2, we calculate the F value of the first-stage regression; conduct the under-identification test; and calculate the partial R-squared between the instrumental variable and the dependent variable. These tests can support the validity of city average commute time as the instrumental variable.

### 3.2. Hourly Wage and Commute Time

Table 3 presents estimated results for the effect of commute time on hourly wage. The result of the fixed effect model, shown in column (1) of Table 3, demonstrates that commute time does not have a significant effect on wage. According to column (2) of Table 3, even when city average commute time is adopted to correct the endogeneity problem of individual commute time, such an effect is still insignificant. Because health status may have an effect on the worker’s productivity, in column (3) we include several health conditions, including stroke, fracture, high blood pressure, diabetes, and myocardial infarction. However, estimation results do not show a significant effect of commute time on hourly wage, which is different from the findings in theoretical derivations.

In addition, cigarette smoking has a negative effect on hourly wage since smoking can impact health and then reduce labor productivity. Even when more health variables are included in the regression, the cigarette variable still has a significant and large negative effect on wage. The labor market demand side variable measured by the ratio of non-public employment in the city, city human capital level, and labor market conditions measured by job changed ratio in the city do not have a significant effect on hourly wage.

### 3.3. Second-Stage Results for Daily Labor Supply

In the second stage, we studied how commute time affects labor supply, in which three variables were used to measure labor supply, that is, daily workhours, workdays per week, and weekly workhours.

The fixed effect model in column (1) of Table 4 shows that commute time has a positive relationship with working hours per day. As commute time may be endogenous, in column (2) of Table 4, we adopt city average commute time as an instrumental variable to correct the endogeneity problem. Result shows that commute time insignificantly decreases daily workhours. The family income and asset variables may be endogenous. Compared with column (2), we control the asset and other family members’ income variables additionally in column (2). We find that the effect of commute time on daily labor supply, that is, the coefficient of interest, changes little when we control these two variables. Based on this result, although there might exist an endogeneity problem of the family income and asset variables, given the very limited impacts on the magnitude and significance of the coefficient of interest, the inclusion of asset and other family members’ income variables as control variables might not be an important issue.

Because commute time does not have any significant relationship with hourly wage, as shown in Section 3.2, we can infer that in labor supply regressions controlling wage additionally will not change the value and significance level of commute time. The regression result in column (4) supports this claim. Since wage and labor supply are simultaneously determined in labor market, the endogeneity problem in terms of hourly wage is still an issue. Column (5) in Table 4, in which the interaction term of experience and age is adopted as an instrumental variable for hourly wage, shows that commute time still has insignificant effect on daily workhours. An interesting result is the positive effect of the number of cigarettes on daily labor supply. One possible explanation is that the cigarette variable represents the workers’ occupations. For example, blue collar workers work more hours and are also more likely to smoke. However, this explanation is rejected by the result that controlling occupations additionally does not alter the value and significance level of the cigarette variable. A second explanation is that the cigarette variable represents ambition and a smoking person has strong incentive to work.

### 3.4. Second-Stage Results for Workdays per Week

The second measurement for labor supply is workdays per week. For workers’ daily labor supply, commute time is fixed cost. However, for weekly labor supply, commute time is a variable cost. Given total weekly workhours, workers can reduce workdays per week and increase daily labor supply to minimize cost. Even when we relax the assumption that total weekly workhours are constant, workers can still reduce cost by decreasing workdays.

Table 5 displays regression results for workdays per week. When city average commute time is used as an instrumental variable to correct individual commute time’s endogeneity problem, the coefficient of individual commute time changes from an insignificant positive effect to a significant negative effect. Even when we control the family income and asset variables, such a result is still valid. Controlling hourly wage additionally, the coefficient of commute time does not change. Adopting the interaction term of experience and age to correct bias caused by the endogeneity of hourly wage, the coefficient of commute time is still unchanged. To summarize, a 1% increase in commute time will significantly decrease workdays per week by 0.0735%.

### 3.5. Second-Stage Results for Weekly Workhours

Weekly workhours are defined as the product of daily workhours and workdays per week. As we took the logarithm in three dependent variables in terms of labor supply, the coefficients in weekly workhours regression are equal to the sum of coefficients in the daily workhours and workdays per week regressions.

According to Table 6, the elasticity of weekly workhours with respect to commute time is −0.107. This means that when individual commute time increases 1%, workers will work 0.107% less hours in a week. The cigarette variable has a positive effect on weekly workhours. Family income and asset variables have insignificant effects on weekly workhours. Controlling these two variables additionally does not alter the value and significance level of individual commute time. City characteristics, that is, city average education attainment, employment change ratio, and non-public employment rate, do not have effects on individual weekly workhours. Even by adopting the interaction term of experience and age as an instrumental variable to correct the hourly wage’s endogeneity problem, such a causal effect does not change.

### 3.6. Additional Results: Job Changed and Unchanged Workers

There are three possibilities that can explain workers reporting a change in commute time. The first possibility is the change of residential or work locations. In the CHNS subsample we selected, residential location was fixed. Therefore, a change of work location will alter commute time. The second is the change of transportation conditions in the city. Better transportation conditions reduce individual commute time. Third, commute time is self-reported and may thus vary due to measurement errors.

We separated the data based on whether respondents changed their jobs since the previous survey. When individuals find new jobs, they can simultaneously choose their work location, wage, and commute time to maximize their utility. Proposition 1 proposes theoretically that, for individuals who change jobs, longer commute time will be compensated by a higher wage. For individuals who do not change jobs, there is no causal relationship between wage and commute time. In both cases, commute time can have positive or negative effects on labor supply.

Table 7 shows the labor supply and wage regression results for the subsample of job changed workers. Table 8 presents results for job unchanged workers.

Commute time has a positive effect on hourly wage for job changed workers. However, this effect is insignificant. In contrast, commute time has an insignificant negative effect on the hourly wage for job unchanged workers.

For individuals who change jobs, commute time decreases daily workhours and weekly workhours insignificantly, while it significantly decreases workdays per week. Furthermore, the effects are larger than those in the full sample. For example, in the full sample, the elasticity for workdays per week is −0.0735, while it is −0.243 for the job changed subsample.

For individuals who do not change job, individual commute time is insignificant in four regressions and the absolute values are also less than those in the subsample of job changed workers.

### 3.7. Additional Results: Male and Female Workers

Since females do more household work than males in China, commute time may have heterogeneous effects on labor supply for males and females. Table 9 and Table 10 show results for job changed and unchanged workers, respectively, in which the interaction term of commute time and female dummy is controlled. Since this interaction term has an endogeneity problem, the interaction term of city average commute time and female dummy is used as an instrumental variable. Both tables show that gender does not play a role in the effect of commute time on labor supply.

## 4. Discussions

One of the main findings of this paper is that commute time does not have significant effect on daily workhours but significantly reduces workdays per week and weekly workhours. This result is different from findings in Germany and Spain, in which commute time significantly increases daily workhours. In addition, our results also differ from the findings in Germany that show commute time slightly increases weekly workhours but does not affect the number of workdays.

One reason for the different patterns might relate to the methodology. The study for Germany considers an employer-induced change in workplace location and thus uses an exogeneous individual commute time. In the study for Spain, the endogeneity of individual commute time is corrected by using the prices of an individual’s house. Given the availability of the data, in this paper, we use the city average commute time as the instrumental variable to correct the endogeneity. Although we employ a distinct methodology compared with the studies for Germany and Spain, given the discussion on the instrumental variable in this paper, our methodology is valid and effective for the understanding of the causal effect of commute time. Consequently, the different methodologies might not be the main reason for the heterogeneous effects of commute time on daily labor supply found in Germany, Spain, and China.

Another reason might be the different degrees of competition intensity among workers in the labor market. In Germany and Spain, the competition intensity among workers is relatively low compared to China. In such circumstances, workers might have stronger bargaining power and accordingly have the possibility to discuss the number of working hours when they sign a working contract with employers. If workers have to suffer a longer commute time, they may choose to work longer in a working day and work fewer days in a week in order to lower the total cost of the commute time in a week. In this scenario, the longer commute time may increase the daily workhours, which is the case in Germany and Spain. However, the competition among workers is more intense in China than Germany and Spain. Workers in China have very limited bargaining power and have to follow job requirements strictly according to the willingness of employers. Thus, we may observe in China that a longer commute time of workers does not significantly affect the daily workhours. In addition, as workers have more flexibility in choosing a job with the desired number of working days in China, a longer commute time may decrease the number of working days in a week and hence the number of weekly workhours.

According to our preliminary insights about how commute time affects labor supply in urban China, we can identify the following policy implications for the promotion of active commuting. In Germany and Spain, as an increase in commute time has positive effects on labor supply, the economic consequences of active commuting will not be a big issue. However, as the weekly labor supply will be reduced by a longer commute time in urban China, the promotion of active commuting might be detrimental to the economic development to some extent. Therefore, local governments in China should invest more in transportation infrastructure, for instance, building more exclusive bike lanes and sidewalks. In this way, better infrastructure can not only encourage people to choose active commuting, but also shorten the commute time effectively and mitigate the potential adverse effects of active commuting.

Further, because we observe that the negative effect of commute time is particularly strong and significant for job changed workers, it might be more efficient to give priority to the infrastructure investments mentioned above in urban areas where house rental is more active.

## 5. Conclusions

This paper investigates the impact of commute time on labor supply in urban China and discusses the implications for the promotion of active commuting. Based on the CHNS panel data, we measure labor supply by daily workhours, workdays per week, and weekly workhours, using the analytical framework of combining a fixed effect model and instrumental variables. The main findings can be summarized as follows.

First, in contrast with existing empirical evidence from Germany and Spain, the effect of commute time on daily labor supply is insignificant in urban China. Furthermore, commute time decreases workdays per week and weekly workhours. These distinct patterns might closely relate to the different degrees of competition intensity among workers in labor markets. Second, commute time does not have a significant effect on labor supply for job unchanged workers, while the effect of commute time on workdays per week is large and statistically significant for job changed workers. Since job changed workers can choose a new job with different job characteristics, commute time will have a strong effect. Third, there is no statistically significant difference in the effects of commute time on labor supply between males and females. In addition, smoking is found to have a negative effect on hourly wage and positive effects on daily and weekly workhours. The former may relate to the fact that smoking can impact health and then reduce labor productivity, while the latter may relate to the possibility that smoking represents ambition and a smoking person has a strong incentive to work. Finally, policy implications for promoting active commuting are discussed. 

## Figures and Tables

**Table 1:** Descriptive statistics.

	**2004**		**2006**	
	**Mean**	**S.D.**	**Mean**	**S.D.**
Daily workhours	8.05	1.37	7.93	1.24
Workdays per week	5.51	0.82	5.43	0.85
Weekly workhours	44.56	11.75	43.30	10.93
Hourly wage	8.42	7.28	11.16	15.91
Commute time	29.17	25.00	34.04	25.86
Family wealth	85,218.59	223,544.99	68,030.82	179,556.20
Other family members’ income	67,335.48	51,489.82	71,479.22	57,398.39
Age	39.86	9.38	41.05	9.47
Cigarette	0.32	0.47	0.33	0.47
Number of children less than 6 years old	0.17	0.38	0.15	0.37
Number of family members	3.22	1.15	3.10	1.09
City job change rate	0.16	0.09	0.11	0.07
City average education	11.07	0.96	11.45	1.13
City non-public employment rate	0.29	0.15	0.37	0.13
City average commute time	29.17	6.15	34.04	7.29
*N*	604		693	
	**2009**		**2011**	
	**Mean**	**S.D.**	**Mean**	**S.D.**
Daily workhours	8.02	1.47	8.11	1.37
Workdays per week	5.44	0.84	5.54	0.89
Weekly workhours	43.71	11.19	45.10	11.59
Hourly wage	13.90	13.75	15.54	17.93
Commute time	36.09	26.11	34.68	25.84
Family wealth	298,918.15	325,010.84	941,293.65	1,665,275.68
Other family members’ income	93,313.23	70,074.26	110,297.02	98,893.49
Age	41.86	9.53	42.74	9.10
Cigarette	0.34	0.47	0.34	0.47
Number of children less than 6 years old	0.16	0.40	0.14	0.37
Number of family members	3.21	1.23	3.27	1.27
City job change rate	0.15	0.08	0.14	0.08
City average education	11.29	1.24	11.24	1.22
City non-public employment rate	0.43	0.18	0.44	0.17
City average commute time	36.09	5.82	34.68	6.50
*N*	775		655	

**Table 2:** Effect of city average commute time on individual commute time.

VARIABLES	(1)	(2)	(3)
	Dependent Variable: Log (Individual Commute Time)		
Log (City average commute time)	0.875 ***	0.920 ***	0.920 ***
	(0.131)	(0.133)	(0.133)
Log (Hourly wage)			−0.0101
			(0.0365)
Cigarette	−0.0638	−0.0625	−0.0637
	(0.0638)	(0.0640)	(0.0644)
Age	0.00844	0.0141	0.0148
	(0.00920)	(0.0107)	(0.0109)
Number of children less than 6 years old	0.0864	0.0917	0.0924
	(0.0626)	(0.0626)	(0.0627)
Number of family members	0.00289	0.00184	0.000963
	(0.0291)	(0.0291)	(0.0295)
Log (Asset)	−0.0126	−0.0121	−0.0120
	(0.0123)	(0.0123)	(0.0123)
Log (Other family members’ income)	0.0553 **	0.0550 **	0.0580 **
	(0.0272)	(0.0273)	(0.0289)
Stroke	−0.864 *	−0.859 *	−0.856 *
	(0.497)	(0.499)	(0.500)
Fracture	−0.00158	−0.00491	−0.00422
	(0.0771)	(0.0768)	(0.0769)
Blood pressure	0.153 *	0.152 *	0.150 *
	(0.0860)	(0.0867)	(0.0863)
Diabetes	0.0934	0.0969	0.101
	(0.165)	(0.163)	(0.163)
Myocardial infarction	0.713	0.672	0.670
	(0.577)	(0.600)	(0.601)
City job change rate		−0.107	−0.107
		(0.238)	(0.239)
City average education		−0.00749	−0.00743
		(0.0359)	(0.0359)
City non-public employment rate		−0.324	−0.324
		(0.204)	(0.204)
Constant	−0.686	−0.854	−0.893
	(0.471)	(0.580)	(0.592)
Observations	2727	2727	2727
R-squared	0.652	0.653	0.653
Number of id	1077	1077	1077
Year FE	YES	YES	YES
F value	44.907	47.978	47.923
Under-identification test	45.164	48.306	48.268
Partial R-squared between IV and dependent variable	0.017	0.017	0.017

Note: Coefficients of year fixed effect are not reported; Standard errors adjusted for clustering around individuals are in parentheses; *** *p* < 0.01, ** *p* < 0.05, * *p* < 0.1.

**Table 3:** Hourly wage regression results.

	(1)	(2)	(3)
	Dependent Variable: Log (Hourly Wage)		
VARIABLES	FE	FE + 2SLS	FE + 2SLS
Log (Commute time)	0.00423	−0.0475	−0.0544
	(0.0193)	(0.109)	(0.109)
Cigarette	−0.116 *	−0.118 *	−0.118 *
	(0.0629)	(0.0632)	(0.0623)
Experience	0.00596	0.00471	0.00550
	(0.0143)	(0.0145)	(0.0144)
Experience square	−0.000398 *	−0.000377	−0.000389 *
	(0.000226)	(0.000231)	(0.000231)
City job change rate	0.0612	0.0779	0.0737
	(0.169)	(0.170)	(0.171)
City average education	0.00455	0.00438	0.00613
	(0.0271)	(0.0272)	(0.0274)
City non-public employment rate	−0.0609	−0.0733	−0.0794
	(0.160)	(0.162)	(0.159)
Stroke			0.280
			(0.383)
Fracture			0.0449
			(0.0424)
Blood pressure			−0.179 **
			(0.0761)
Diabetes			0.393 ***
			(0.112)
Myocardial infarction			−0.184 *
			(0.109)
Constant	1.924 ***	2.101 ***	2.095 ***
	(0.406)	(0.577)	(0.573)
Observations	2727	2727	2727
R-squared	0.766	0.766	0.769
Number of id	1077	1077	1077
Year FE	YES	YES	YES

Note: Coefficients of year fixed effect are not reported; Standard errors adjusted for clustering around individuals are in parentheses; *** *p* < 0.01, ** *p* < 0.05, * *p* < 0.1.

**Table 4:** Effect of commute time on labor supply—daily workhours.

	(1)	(2)	(3)	(4)	(5)
	Dependent Variable: Log (Daily Workhours)				
VARIABLES	FE	FE + 2SLS	FE + 2SLS	FE + 2SLS	FE + 2SLS
Log (Commute time)	0.00259	−0.0306	−0.0332	−0.0332	−0.0333
	(0.00675)	(0.0377)	(0.0377)	(0.0364)	(0.0418)
Log (Hourly wage)				−0.138 ***	0.107
				(0.0179)	(0.194)
Cigarette	0.0555 **	0.0538 **	0.0529 **	0.0366 *	0.0656 *
	(0.0228)	(0.0228)	(0.0226)	(0.0190)	(0.0375)
Age	−0.00118	−4.23 × 10^−6^	0.000773	0.0105 ***	−0.00670
	(0.00211)	(0.00258)	(0.00270)	(0.00289)	(0.0139)
Number of children less than 6 years old	−0.0441 ***	−0.0419 **	−0.0408 **	−0.0312 *	−0.0481 *
	(0.0166)	(0.0167)	(0.0171)	(0.0159)	(0.0252)
Number of family members	−0.00443	−0.00383	−0.00479	−0.0168 **	0.00448
	(0.00748)	(0.00756)	(0.00761)	(0.00745)	(0.0186)
City job change rate	0.0330	0.0441	0.0452	0.0460	0.0445
	(0.0618)	(0.0630)	(0.0629)	(0.0602)	(0.0695)
City average education	0.00170	0.00159	0.00114	0.00199	0.000485
	(0.00997)	(0.0100)	(0.0101)	(0.00971)	(0.0112)
City non-public employment rate	−0.0271	−0.0346	−0.0354	−0.0443	−0.0286
	(0.0597)	(0.0624)	(0.0625)	(0.0573)	(0.0716)
Log (Asset)			−0.00276	−0.00119	−0.00397
			(0.00328)	(0.00309)	(0.00447)
Log (Other family members’ income)			0.00415	0.0443 ***	−0.0268
			(0.00919)	(0.0111)	(0.0581)
Constant	2.105 ***	2.162 ***	2.132 ***	1.609 ***	2.535 ***
	(0.110)	(0.129)	(0.140)	(0.154)	(0.748)
Observations	2727	2727	2727	2727	2727
R-squared	0.563	0.563	0.563	0.615	0.563
Number of id	1077	1077	1077	1077	1077
Health conditions	YES	YES	YES	YES	YES
Year FE	YES	YES	YES	YES	YES

Note: Columns (2), (3), (4), and (5) adopt city average commute time as instrumental variable for individual commute time; Column (5) additionally adopts the interaction term of experience and age as instrumental variable for hourly wage; Coefficients of year fixed effect and health conditions are not reported; Standard errors adjusted for clustering around individuals are in parentheses; *** *p* < 0.01, ** *p* < 0.05, * *p* < 0.1.

**Table 5:** Effect of commute time on labor supply—workdays per week.

	(1)	(2)	(3)	(4)	(5)
	Dependent Variable: Log (Workdays per Week)				
VARIABLES	FE	FE + 2SLS	FE + 2SLS	FE + 2SLS	FE + 2SLS
Log (Commute time)	0.00316	−0.0702 *	−0.0735 *	−0.0735 *	−0.0735 *
	(0.00606)	(0.0407)	(0.0416)	(0.0395)	(0.0410)
Log (Hourly wage)				−0.123 ***	−0.0208
				(0.0162)	(0.132)
Cigarette	0.0315 *	0.0277	0.0264	0.0118	0.0240
	(0.0185)	(0.0190)	(0.0192)	(0.0172)	(0.0263)
Age	−0.00210	0.000482	0.00132	0.00994 ***	0.00278
	(0.00166)	(0.00225)	(0.00268)	(0.00301)	(0.0100)
Number of children less than 6 years old	0.00778	0.0127	0.0146	0.0231	0.0161
	(0.0151)	(0.0165)	(0.0170)	(0.0166)	(0.0189)
Number of family members	−0.00475	−0.00343	−0.00536	−0.0161 **	−0.00717
	(0.00694)	(0.00721)	(0.00749)	(0.00762)	(0.0135)
City job change rate	−0.00852	0.0161	0.0172	0.0180	0.0173
	(0.0562)	(0.0597)	(0.0602)	(0.0563)	(0.0591)
City average education	−0.00858	−0.00882	−0.00951	−0.00875	−0.00938
	(0.0101)	(0.0106)	(0.0106)	(0.00993)	(0.0106)
City non-public employment rate	0.0670	0.0503	0.0494	0.0415	0.0480
	(0.0517)	(0.0536)	(0.0539)	(0.0499)	(0.0533)
Log (Asset)			−0.00379	−0.00239	−0.00355
			(0.00301)	(0.00279)	(0.00319)
Log (Other family members’ income)			0.00818	0.0439 ***	0.0142
			(0.00799)	(0.00987)	(0.0401)
Constant	1.844 ***	1.970 ***	1.912 ***	1.447 ***	1.833 ***
	(0.117)	(0.141)	(0.141)	(0.132)	(0.528)
Observations	2727	2727	2727	2727	2727
R-squared	0.563	0.568	0.568	0.625	0.568
Number of id	1077	1077	1077	1077	1077
Health conditions	YES	YES	YES	YES	YES
Year FE	YES	YES	YES	YES	YES

Note: Columns (2), (3), (4), and (5) adopt city average commute time as an instrumental variable for individual commute time; Column (5) additionally adopts the interaction term of experience and age as an instrumental variable for hourly wage; Coefficients of year fixed effect and health conditions are not reported; Standard errors adjusted for clustering around individuals are in parentheses; *** *p* < 0.01, ** *p* < 0.05, * *p* < 0.1.

**Table 6:** Effect of commute time on labor supply—weekly workhours.

	(1)	(2)	(3)	(4)	(5)
	Dependent Variable: Log (Weekly Workhours)				
VARIABLES	FE	FE+2SLS	FE+2SLS	FE+2SLS	FE+2SLS
Log (Commute time)	0.00575	−0.101 *	−0.107 *	−0.107 **	−0.107 *
	(0.00937)	(0.0574)	(0.0585)	(0.0537)	(0.0624)
Log (Hourly wage)				−0.261 ***	0.0858
				(0.0243)	(0.249)
Cigarette	0.0871 ***	0.0815 ***	0.0794 **	0.0484 **	0.0896 *
	(0.0307)	(0.0310)	(0.0309)	(0.0237)	(0.0475)
Age	−0.00328	0.000477	0.00209	0.0204 ***	−0.00392
	(0.00273)	(0.00348)	(0.00399)	(0.00418)	(0.0181)
Number of children less than 6 years old	−0.0363 *	−0.0291	−0.0261	−0.00802	−0.0321
	(0.0217)	(0.0233)	(0.0239)	(0.0215)	(0.0320)
Number of family members	−0.00918	−0.00725	−0.0101	−0.0329 ***	−0.00269
	(0.0108)	(0.0113)	(0.0114)	(0.0107)	(0.0246)
City job change rate	0.0245	0.0602	0.0623	0.0640	0.0618
	(0.0873)	(0.0903)	(0.0908)	(0.0818)	(0.0973)
City average education	−0.00688	−0.00723	−0.00837	−0.00677	−0.00890
	(0.0146)	(0.0152)	(0.0153)	(0.0138)	(0.0166)
City non-public employment rate	0.0399	0.0157	0.0140	−0.00286	0.0195
	(0.0807)	(0.0867)	(0.0870)	(0.0740)	(0.0957)
Log (Asset)			−0.00655	−0.00358	−0.00753
			(0.00474)	(0.00421)	(0.00583)
Log (Other family members’ income)			0.0123	0.0882***	−0.0126
			(0.0125)	(0.0148)	(0.0741)
Constant	3.950 ***	4.132 ***	4.043 ***	3.056 ***	4.368 ***
	(0.167)	(0.201)	(0.212)	(0.210)	(0.977)
Observations	2727	2727	2727	2727	2727
R-squared	0.568	0.577	0.578	0.676	0.578
Number of id	1077	1077	1077	1077	1077
Health conditions	YES	YES	YES	YES	YES
Year FE	YES	YES	YES	YES	YES

Note: Columns (2), (3), (4), and (5) adopt city average commute time as an instrumental variable for individual commute time; Column (5) additionally adopts the interaction term of experience and age as an instrumental variable for hourly wage; Coefficients of year fixed effect and health conditions are not reported; Standard errors adjusted for clustering around individuals are in parentheses; *** *p* < 0.01, ** *p* < 0.05, * *p* < 0.1.

**Table 7:** Labor supply and wage for job changed workers.

VARIABLES	(1)	(2)	(3)	(4)
	Log (Daily	Log (Workdays	Log (Weekly	Log (Hourly
	Workhours)	per Week)	Workhours)	Wage)
Log (Commute time)	−0.0544	−0.243 *	−0.298	0.234
	(0.160)	(0.127)	(0.242)	(0.328)
Cigarette	0.0119	−0.0625	−0.0506	−0.0442
	(0.0863)	(0.0891)	(0.156)	(0.250)
Age	−0.00751	−0.00560	−0.0131	
	(0.00875)	(0.0107)	(0.0165)	
Number of children less than 6 years old	−0.0771	−0.0758	−0.153	
	(0.0853)	(0.0601)	(0.117)	
Number of family members	−0.0336	0.0412	0.00756	
	(0.0310)	(0.0341)	(0.0501)	
Log (Asset)	0.00228	−0.000458	0.00183	
	(0.0160)	(0.0140)	(0.0234)	
Log (Other family members’ incomes)	−0.0459	0.0395	−0.00635	
	(0.0391)	(0.0379)	(0.0629)	
City job change rate	−0.172	−0.145	−0.317	0.949
	(0.313)	(0.354)	(0.529)	(0.820)
City average education	−0.0153	−0.0677	−0.0830	0.0627
	(0.0414)	(0.0486)	(0.0728)	(0.128)
City non-public employment rate	0.163	0.399	0.562	−0.394
	(0.256)	(0.261)	(0.453)	(0.497)
Experience				0.0790
				(0.0569)
Experience square				−0.00127
				(0.000825)
Constant	3.288 ***	2.776 ***	6.065 ***	−0.664
	(0.765)	(0.871)	(1.284)	(2.562)
Observations	296	296	296	296
R-squared	0.695	0.607	0.643	0.761
Number of id	139	139	139	139
Health conditions	YES	YES	YES	YES
Year FE	YES	YES	YES	YES

Note: Columns (1), (2), (3), and (4) adopt city average commute time as an instrumental variable for individual commute time; Column (4) additionally adopts the interaction term of experience and age as an instrumental variable for hourly wage; Coefficients of year fixed effect and health conditions are not reported; Standard errors adjusted for clustering around individuals are in parentheses; *** *p* < 0.01, ** *p* <0.05, * *p* < 0.1.

**Table 8:** Labor supply and wage for job unchanged workers.

	(1)	(2)	(3)	(4)
	Log (Daily	Log (Workdays	Log (Weekly	Log (Hourly
VARIABLES	Workhours)	Per Week)	Workhours)	Wage)
Log (Commute time)	−0.0140	−0.0465	−0.0605	−0.0713
	(0.0352)	(0.0444)	(0.0564)	(0.112)
Cigarette	0.0620 ***	0.0340 *	0.0960 ***	−0.122 *
	(0.0238)	(0.0183)	(0.0290)	(0.0648)
Age	0.000842	−9.73 × 10^−5^	0.000745	
	(0.00283)	(0.00294)	(0.00405)	
Number of children less than 6 years old	−0.0334 **	0.0187	−0.0147	
	(0.0148)	(0.0183)	(0.0218)	
Number of family members	−0.00210	−0.0131 *	−0.0152	
	(0.00740)	(0.00697)	(0.0107)	
Log (Asset)	−0.00175	−0.00330	−0.00505	
	(0.00326)	(0.00308)	(0.00468)	
Log (Other family members’ incomes)	0.00751	0.00588	0.0134	
	(0.00959)	(0.00787)	(0.0125)	
City job change rate	0.0721	0.00565	0.0778	0.0573
	(0.0624)	(0.0604)	(0.0893)	(0.175)
City average education	0.000592	−0.00572	−0.00513	0.00260
	(0.0100)	(0.0109)	(0.0151)	(0.0288)
City non-public employment rate	−0.0681	0.0374	−0.0307	−0.0813
	(0.0640)	(0.0548)	(0.0868)	(0.170)
Experience				0.00261
				(0.0153)
Experience square				−0.000370
				(0.000249)
Constant	2.017 ***	1.884 ***	3.901 ***	2.275 ***
	(0.130)	(0.139)	(0.196)	(0.578)
Observations	2492	2492	2492	2492
R-squared	0.570	0.578	0.591	0.773
Number of id	990	990	990	990
Health conditions	YES	YES	YES	YES
Year FE	YES	YES	YES	YES

Note: Columns (1), (2), (3), and (4) adopt city average commute time as an instrumental variable for individual commute time; Column (4) additionally adopts the interaction term of experience and age as an instrumental variable for hourly wage; Coefficients of year fixed effect and health conditions are not reported; Standard errors adjusted for clustering around individuals are in parentheses; *** *p* < 0.01, ** *p* <0.05, * *p* < 0.1.

**Table 9:** Labor supply and wage for job changed workers based on gender.

	(1)	(2)	(3)	(4)
	Log (Daily	Log (Workdays	Log (Weekly	Log (Hourly
VARIABLES	Workhours)	per Week)	Workhours)	Wage)
Log (Commute time) × Female dummy	0.00278	0.0183	0.0210	0.138
	(0.170)	(0.179)	(0.293)	(0.381)
Log (Commute time)	−0.0559	−0.253	−0.309	0.158
	(0.202)	(0.169)	(0.320)	(0.435)
Cigarette	0.0113	−0.0666	−0.0553	−0.0713
	(0.103)	(0.103)	(0.182)	(0.269)
Age	−0.00756	−0.00591	−0.0135	
	(0.00922)	(0.0113)	(0.0178)	
Number of children less than 6 years old	−0.0769	−0.0745	−0.151	
	(0.0829)	(0.0621)	(0.118)	
Number of family members	−0.0337	0.0410	0.00728	
	(0.0314)	(0.0338)	(0.0506)	
Log (Asset)	0.00233	−0.000140	0.00219	
	(0.0167)	(0.0152)	(0.0246)	
Log (Other family members’ incomes)	−0.0455	0.0421	−0.00336	
	(0.0450)	(0.0465)	(0.0751)	
City job change rate	−0.172	−0.141	−0.313	0.979
	(0.308)	(0.353)	(0.523)	(0.823)
City average education	−0.0153	−0.0678	−0.0831	0.0628
	(0.0415)	(0.0489)	(0.0731)	(0.127)
City non-public employment rate	0.163	0.399	0.562	−0.388
	(0.257)	(0.261)	(0.453)	(0.500)
Experience				0.0708
				(0.0657)
Experience square				−0.00117
				(0.000921)
Constant	3.286 ***	2.760 ***	6.046 ***	−0.530
	(0.752)	(0.870)	(1.259)	(2.707)
Observations	296	296	296	296
R-squared	0.695	0.607	0.643	0.761
Number of id	139	139	139	139
Health conditions	YES	YES	YES	YES
Year FE	YES	YES	YES	YES

Note: Columns (1), (2), (3), and (4) adopt city average commute time as an instrumental variable for individual commute time; Column (4) additionally adopts the interaction term of experience and age as an instrumental variable for hourly wage; Coefficients of year fixed effect and health conditions are not reported; Standard errors adjusted for clustering around individuals are in parentheses; *** *p* < 0.01, ** *p* < 0.05, * *p* < 0.1.

**Table 10:** Labor supply and wage for job unchanged workers based on gender.

	(1)	(2)	(3)	(4)
	Log (Daily	Log (Workdays	Log (Weekly	Log (Hourly
VARIABLES	Workhours)	per Week)	Workhours)	Wage)
Log (Commute time) × Female dummy	−0.0396	0.0175	−0.0220	0.0187
	(0.0563)	(0.0511)	(0.0803)	(0.164)
Log (Commute time)	0.00438	−0.0547	−0.0503	−0.0799
	(0.0462)	(0.0590)	(0.0730)	(0.144)
Cigarette	0.0617 ***	0.0341 *	0.0958 ***	−0.122 *
	(0.0238)	(0.0183)	(0.0290)	(0.0648)
Age	0.000704	−3.61 × 10^−5^	0.000668	
	(0.00289)	(0.00299)	(0.00413)	
Number of children less than 6 years old	−0.0347 **	0.0193	−0.0154	
	(0.0150)	(0.0187)	(0.0222)	
Number of family members	−0.00285	−0.0128 *	−0.0156	
	(0.00752)	(0.00706)	(0.0109)	
Log (Asset)	−0.00167	−0.00334	−0.00500	
	(0.00329)	(0.00310)	(0.00472)	
Log (Other family members’ incomes)	0.00763	0.00583	0.0135	
	(0.00960)	(0.00783)	(0.0125)	
City job change rate	0.0654	0.00865	0.0740	0.0604
	(0.0647)	(0.0608)	(0.0906)	(0.178)
City average education	0.000185	−0.00554	−0.00535	0.00274
	(0.00999)	(0.0110)	(0.0151)	(0.0289)
City non-public employment rate	−0.0679	0.0373	−0.0306	−0.0812
	(0.0643)	(0.0549)	(0.0869)	(0.170)
Experience				0.00239
				(0.0154)
Experience square				−0.000369
				(0.000249)
Constant	2.023 ***	1.881 ***	3.905 ***	2.279 ***
	(0.130)	(0.139)	(0.196)	(0.580)
Observations	2492	2492	2492	2492
R-squared	0.570	0.578	0.591	0.773
Number of id	990	990	990	990
Health conditions	YES	YES	YES	YES
Year FE	YES	YES	YES	YES

Note: Columns (1), (2), (3), and (4) adopt city average commute time as an instrumental variable for individual commute time; Column (4) additionally adopts the interaction term of experience and age as an instrumental variable for hourly wage; Coefficients of year fixed effect and health conditions are not reported; Standard errors adjusted for clustering around individuals are in parentheses; *** *p* < 0.01, ** *p* < 0.05, * *p* < 0.1.

## References

[1] Gutiérrez-i-Puigarnau E., van Ommeren J.N. (2010). Labor supply and commuting. J. Urban Econ..

[2] Van Ommeren J.N., Gutiérrez-i-Puigarnau E. (2011). Are workers with a long commute less productive? An empirical analysis of absenteeism. Reg. Sci. Urban Econ..

[3] (2011). Commuting Time and Labor Supply: A Causal Effect?. https://www.iza.org/publications/dp/5529/commuting-time-and-labour-supply-a-causal-effect.

[4] Cogan J.F. (1981). Fixed costs and labor supply. Econometrica.

[5] Parry I.W.H., Bento A. (2001). Revenue recycling and the welfare effects of road pricing. Scand. J. Econ..

[6] Gershenson S. (2013). The causal effect of commute time on labor supply: Evidence from a natural experienceeriment involving substitute teachers. Transp. Res. Part A Policy Pract..

[7] Black D.A., Kolesnikova N., Taylor L.J. (2014). Why do so few women work in New York (and so many in Minneapolis)? Labor supply of married women across US cities. J. Urban Econ..

[8] White M.J. (1988). Location choice and commuting behavior in cities with decentralized employment. J. Urban Econ..

[9] Hamilton B.W. (1989). Wasteful commuting again. J. Political Econ..

[10] Hamilton B.W., Röell A. (1982). Wasteful commuting. J. Political Econ..

[11] Small K.A., Song S. (1992). “Wasteful” commuting: A resolution. J. Political Econ..

[12] White M.J. (1988). Urban commuting journeys are not “wasteful”. J. Political Econ..

